# Family dermatology life quality index (F-DLQI): German validation and applicability to parents of infants and toddlers with dermatological conditions

**DOI:** 10.1007/s00431-025-06494-x

**Published:** 2025-10-20

**Authors:** Juliane Traxler, Neuza da Silva Burger, Matthias Augustin, Hagen Ott, Sanna Hoffmann, Regina Fölster-Holst, Maria Baumeister, Petra Staubach, Rachel Sommer

**Affiliations:** 1 German Center for Health Services Research in Dermatology (CVderm), Institute for Health Services Research in Dermatology and Nursing (IVDP), University Medical Center Hamburg-Eppendorf (UKE), Martinistraße 52, 20246 Hamburg, Germany; 2 Section of Integrated Pediatric Dermatology, Center for Children With Medical and Developmental Complexity, Munich University, Ludwig-Maximilians-University Hospital, Munich, Germany; 3 Clinic for Dermatology, Venereology and Allergology, University Medical Center Schleswig-Holstein, Kiel, Germany; 4 Specialist Clinic Gaißach (Rehabilitation Clinic for Chronic Diseases in Children, Teenagers and Adults), Gaißach, Germany; 5 Department of Dermatology, University Medical Center, Mainz, Germany; 6 Paediatric Dermatology and Allergology, Children’s Hospital Auf der Bult, Hannover, Germany

**Keywords:** Outcome assessment, Quality of life, Pediatric dermatology, Family, Psychometrics

## Abstract

Parents of children with skin conditions face an additional caregiving burden and significant health-related quality of life (HRQoL) impairments. This study aimed to (1) test the psychometric properties of the German version of the Family Dermatology Life Quality Index (F-DLQI) in parents of infants and toddlers with any dermatological diagnosis and (2) examine the associations between the parents’ and their children’s HRQoL. Parents (*n* = 126) of 0- to 4-year-old children with any skin disease filled in the F-DLQI and an instrument assessing their children’s HRQoL, the Infants and Toddlers Dermatology Quality of Life questionnaire (InToDermQoL). The attending physician provided clinical information. Internal consistency, 2-week test–retest reliability, measurement error, and known-groups validity across severity levels of the F-DLQI were examined. Associations between the parents’ and their children’s HRQoL were tested using regression analysis. Internal consistency and test–retest reliability of the F-DLQI were good (Cronbach’s *α* = 0.896, ICC = 0.867), and construct validity was confirmed. Parents’ HRQoL (F-DLQI) and children’s current complaints as reported by their parents, but not physician-rated disease severity, were associated with children’s HRQoL. *Conclusion*: The German version of the F-DLQI is a valid and reliable tool in measuring the HRQoL of parents of children ≤ 4 years with dermatological conditions. The majority of the sample had atopic dermatitis, limiting the generalizability of the findings. Future studies need to evaluate its structural validity and responsiveness. Burden experienced by parents should be taken into account by care providers to prevent parental stress from becoming chronic and negatively affecting the child’s HRQoL.

**Table:** 

What is Known:	
• *Pediatric skin diseases can have a great impact on the entire family and impose additional burdensome caregiving demands*	
•* The Family Dermatology Life Quality Index (F-DLQI) is the first dermatology‐specific questionnaire assessing the adverse impact of skin conditions on the health-related quality of life (HRQoL) of patients’ family members*	
What is New:	
*• This is the first cross-cultural adaptation and validation of the F-DLQI for Germany, proving its validity and reliability*	
*• The child’s HRQoL is not only associated with current complaints, like itch and sleeplessness, but also their parents’ HRQoL*

## Introduction

While providing daily care to children is a normative parenting task, parents of children with dermatological conditions face additional, unexpected, and burdensome caregiving demands. These often include time-consuming and expensive treatments, extra housework, sleep disturbances, reduced leisure activities, high rates of work absenteeism or job abandonment, and psychological distress [1, 28]. Several studies reported significant health-related quality of life (HRQoL) impairments and high prevalence of depression and anxiety among parents of children with skin conditions [9, 10]. Because parents play a major role in the management of their child’s skin condition, their HRQoL impairments and mental health problems can significantly impact parent–child interactions and attachment, adherence to treatments, and, ultimately, the child’s HRQoL, development, and wellbeing.

The term “greater patient” has been coined by Basra and Finlay [2] to represent the “secondary” multidimensional impact of skin conditions on patients’ family members. Developed by the same authors, the Family Dermatology Life Quality Index (F-DLQI) [3] was the first dermatology‐specific questionnaire assessing the adverse impact of skin conditions on the HRQoL of patients’ family members. The 10-item questionnaire showed good psychometric performance, including ascertainment of scale unidimensionality, good reliability (with Cronbach’s alpha value of 0.88 and intraclass correlation coefficient [ICC] for test–retest reliability of 0.94), responsiveness to changes in the severity of the patient’s skin condition, and construct validity (with a strong correlation with the patient’s HRQoL impairments; moderate correlation with disease severity; and the ability to discriminate between different dermatoses). The questionnaire has been translated and validated in multiple languages [14, 17, 23, 24], consistently proving its psychometric robustness. To our knowledge, the German version of the F-DLQI has not been psychometrically evaluated.

In the pediatric context, several studies using the F-DLQI have identified significant HRQoL impairments in parents of children diagnosed with different skin diseases, such as atopic dermatitis [7, 18, 21], psoriasis [4, 31], vitiligo [11], or alopecia areata [22]. Consistently, across conditions, the most affected dimensions were emotional and physical wellbeing, burden of care, and household expenditure [4, 11, 18, 21, 31]. Some studies reported significant associations between higher F-DLQI impairments and the child’s female sex [11], female caregiver/mother [18], shorter disease duration [4, 11], greater severity [4, 18, 21, 22], involvement of visible body sites [4], higher treatment expenditure [4], and decreased child’s HRQoL [4, 18]. However, another study found parents’ HRQoL impairments to be independent of the patient’s or caregivers’ characteristics [31].

Most of the existing studies focused on school-aged children [4, 11, 31], and less is known about the impact of caring for an infant/toddler on the parents’ HRQoL. Moreover, the few studies with pre-school children are restricted to atopic dermatitis [7, 18, 21], which can be explained by its high prevalence in the first years of life. The challenging assessment of pediatric HRQoL [5] and the scarcity of dermatology-specific measures for young children with different skin conditions also hinder the examination of associations between pediatric patients’ and their parents’ HRQoL. To address these gaps, the aims of this study are twofold: (1) to test the psychometric performance of the German version of the F-DLQI in parents of children between 0 and 4 years of age with any dermatological condition and (2) to examine the associations between parents’ HRQoL impairments, as assessed by the F-DLQI, and pediatric HRQoL, as assessed by the newly validated German version of the Infants and Toddlers Dermatology Quality of Life (InToDermQoL) questionnaire [6, 8].

## Materials and methods

This study had a cross-sectional observational design and was conducted according to general recommendations for HRQoL measurement [5]. Data collection took place between August 2021 and April 2024. The study was carried out in compliance with the Helsinki Declaration and was approved by the ethics committee of the University Medical Center Hamburg-Eppendorf (reference: LPEK-0304).

### Participants

Parents of an infant or toddler with a skin disease were invited to take part in the survey. Participants were recruited at six German dermatology centers and pediatric hospitals and provided written informed consent prior to the study. Eligibility criteria were checked by the attending physician and included being a parent of a child aged 0–4 years with a diagnosis of a skin disease and having the ability to consent autonomously and to speak and understand German.

### Outcome Measures

Data were collected using pen and paper. The German versions of the following caregiver-reported questionnaires were used.

#### F-DLQI [3]

The F-DLQI is a 10-item generic dermatology-specific questionnaire that assesses the adverse impact of a patient’s skin disease on the HRQoL of family members over the last month, on a scale from 0 (not at all/not relevant) to 3 (very much). The instrument has a recall period of 1 month. The scores of individual items are summed to generate a total score (range 0–30), with higher scores indicating greater impairment of the family member’s HRQoL. The linguistic validation of the F-DLQI for Germany was conducted by the Mapi Research Trust (Lyon, France).

#### InToDermQoL [6]

The InToDermQoL measures the parent-reported HRQoL of infants and toddlers below the age of four, living with any skin disease. It has three versions, with 10, 12, and 15 items, for different age groups of children (< 1 year, 1–2 years, and 3–4 years of age, respectively). The recall period is 1 week, and the items are to be answered on a 4-point scale, ranging from 0 (not at all) to 3 (very). For all children, the total score was computed by calculating the mean score of all items (ranging from 0 to 3), with higher scores indicating more HRQoL impairments.

### Procedure

The attending physician reported on clinical characteristics (diagnosis, current treatment, comorbidities, affected body surface area (BSA; 0–100%), Global Clinical Assessment (GCA)). At baseline (T0), caregivers reported on the sociodemographic information of both themselves (age, relationship to the child) and the child (e.g., age and sex), the child’s current symptoms, the F-DLQI, and InToDermQoL. Respondents were also asked to complete the InToDermQoL and the F-DLQI again 14 days later (T1) and return them to the coordinating study center via an envelope. This time interval was chosen to minimize recall bias while allowing for the assumption that children’s disease activity and its impact on the family would remain stable.

### Statistical Analyses

Statistical analyses were conducted using the Statistical Package for the Social Sciences (SPSS v.27.0; IBM Corp., Armonk, NY). The significance level was established at *p*-value ≤ 0.05. Cases were excluded if two or more items of the F-DLQI were not answered; if one item was left unanswered, it was scored 0, and the total score was computed as usual [3].

All analyses were performed on the baseline data; T1 data were included for the calculation of test–retest reliability and measurement error. Item distribution characteristics including floor and ceiling effects, skewness, and kurtosis were inspected. The *structural validity* of the German F-DLQI was examined using exploratory factor analysis (EFA), as the F-DLQI is based on a reflective model. Common factor analysis was conducted using principal axis factoring with oblimin rotation. Pattern coefficients ≥ 0.3 were considered salient. Cross-loadings were rejected to achieve simple structure [27]. Factors with at least three salient loadings, internal consistency of *α* ≥ 0.70, which are theoretically meaningful were considered adequate. The *internal consistency* was assessed using Cronbach’s alpha coefficients. To determine the 2-week *test–retest reliability*, Intraclass Correlation Coefficients (ICC; absolute agreement, two-way mixed effects) were calculated [16]. For internal consistency and test–retest reliability, coefficients ≥ 0.50, ≥ 0.75, and ≥ 0.90 were considered moderate, good, and excellent reliability, respectively. To assess whether the sample that completed T1 was different from the total sample, independent *t*-tests were run on the child’s age, age at onset, and BSA, the age of the parent, and the F-DLQI score at T0, and *Χ*^2^-tests were run on categorical variables (child’s sex, GCA, respondent). *Standard errors of measurement* were calculated as $SEm\;=\frac{{SD}_{Difference}}{\surd2}$ [12, 29]. *Minimal detectable change* (MDC) scores were calculated at the 95% confidence interval following the formulas $MDC_{individual}\:=\:SEm\:\times\:1.96\:\times\:\surd2$ and $MDC_{group}\;=\frac{MDC_{individual}}{\sqrt n}$ [12].

*Construct validity* was determined using known-groups validity. ANOVAs were used to test the following a priori-defined hypotheses:

1. Parents of children with moderate to severe disease (GCA ≥ 2) have higher F-DLQI scores than parents of children with no or mild burden (GCA < 2) [4, 18, 21, 22].
2. Parents of infants (0–2 years) have higher F-DLQI scores than parents of toddlers (3–4 years).
3. Parents of girls have higher F-DLQI scores than parents of boys [11].

To identify variables associated with children’s HRQoL, preliminary correlations between InToDermQoL and sociodemographic (child’s sex and age) and clinical (BSA, GCA [< 2 vs ≥ 2], current complaints) characteristics, as well as parental burden (F-DLQI), were calculated. Assumptions for regression analysis were checked, and variables significantly correlating with InToDermQoL scores were entered as independent variables in the regression model.

## Results

In total, 163 families were recruited. Of these, 30 records were incomplete (physician or parent CRF not returned; demographic information on respondent or child not provided), and another seven records had to be excluded because two or more items of the F-DLQI were left unanswered. Hence, 126 children (59% male; mean age = 1.92 ± 1.11 years) were included in the analysis. The majority of the sample had atopic dermatitis (*n* = 107; 85%). Demographic and clinical information of included children and respondents is presented in Table 1.

**Table 1:** Demographics and clinical characteristics [*M* (± SD)] of included children and parents of the total sample and comparison of completers and non-completers of follow-up (T1)

		Total (*n* = 126)	Completers (***n*** = 46)	Non-completers (***n*** = 80)	Comparison between groups
Child					
Gender, *n* (%)	Female	50 (41.0)	18 (39.1)	32 (40.0)	*Χ*^*2*^ = 0.105, *p* = 0.746
	Male	72 (59.0)	28 (60.9)	44 (55.0)	
Age (in years), M ± SD		1.92 (± 1.1)	2.06 (± 1.1)	1.84 (± 1.1)	*t(*124) = 1.045, *p* = 0.298
Age at onset (in years), *M* ± SD		0.56 (± 0.6)	0.43 (± 0.4)	0.64 (± 0.6)	*t*(116.9) = −2.186, *p* = 0.031
Treatment*, *n* (%)	None	2 (1.6)	/	2 (2.5)	
	Topical	114 (91.9)	44 (99.8)	70 (88.6)	
	Systemic	17 (13.7)	9 (20.0)	8 (10.1)	
	Other	13 (10.5)	8 (17.8)	5 (6.3)	
GCA, *n* (%)	1	3 (2.4)	/	3 (4.3)	*Χ*^*2*^ = 2.381, *p* = 0.497
	2	44 (34.9)	19 (43.2)	25 (35.7)	
	3	52 (41.3)	19 (43.2)	33 (47.1)	
	4	15 (11.9)	6 (13.6)	9 (12.9)	
BSA (in %), *M* ± SD		17.96 (± 18.6)	20.71 (± 20.9)	16.16 (± 16.9)	*t*(114) = 1.291, *p* = 0.199
F-DLQI at T0, *M* ± SD		10.02 (± 6.8)	10.89 (± 7.0)	9.53 (± 6.8)	*t*(124) = 1.079, *p* = 0.283
Diagnosis^†^, *n* (%)					
Non-infectious	Atopic dermatitis	107	43	64	
	Hemangioma	7	/	7	
	Irritant diaper dermatitis	3	2	1	
	Alopecia areata	1	/	1	
	Cutaneous mastocytosis	1	1	/	
	Ichthyosis vulgaris	1	/	1	
	Juvenile xanthogranuloma	1	/	1	
	Keratosis pilaris	1	/	1	
	Lichen vidal	1	/	1	
	Lymphangioma	1	/	1	
	Neurofibromatosis	1	/	1	
	Psoriasis	1	/	1	
	Onychodystrophy	1	/	1	
	Seborrheic eczema	1	/	1	
	Tinea amiantacea	1	/	1	
Bacterial infection	Superinfection	3	1	2	
	Impetigo	1	/	1	
Parasite infestation	Scabies	1	1	/	
Fungal infection	Tinea capitis	1	/	1	
Viral infection	Urticaria exanthema	1	/	1	
Respondent, *n* (%)					
Mother		113 (89.7%)	41 (89).1	72 (90.0)	*Χ*^*2*^ = 1.532, *p* = 0.465
Father		11 (8.7%)	5 (10.9)	6 (7.5)	
Both parents		2 (1.6%)	/	2 (2.5)	
Age respondent (in years), *M* ± SD		33.50 (± 5.1)	33.78 (± 5.2)	33.34 (± 5.1)	*t*(110) = 0.438, *p* = 0.662

*GCA* global clinical assessment (one-item measure of dermatosis severity, 1 very mild–mild, 2 mild–moderate, 3 moderate–severe, 4 severe–very severe), *BSA* body surface area

*Some children received more than one type of treatment

†Some children were diagnosed with more than one skin condition

### Psychometric analyses of F-DLQI

No floor or ceiling effects were observed; skewness and kurtosis were within the acceptable range (± 1.5; see Table 2) [25].

**Table 2:** Descriptive statistics of the F-DLQI at baseline and follow-up (T1, *n* = 126)

Item	*M* (SD)	Range	Skewness	Kurtosis	Floor effects	Ceiling effects	Cronbach’s *α*	Item-total correlation	Cronbach’s *α* when item deleted
Full scale	10.02 (6.85)	0–30	0.625	− 0.247	4.0%	0.8%	0.896		
1. Emotional impact	1.50 (0.96)							0.730	0.879
2. Physical well-being	1.49 (1.09)							0.770	0.876
3. Relationships	0.72 (0.94)							0.631	0.886
4. People’s reaction	0.66 (0.87)							0.484	0.895
5. Social life	0.47 (0.84)							0.609	0.888
6. Leisure activities	0.81 (1.00)							0.685	0.882
7. Burden of care	1.72 (0.99)							0.604	0.888
8. Housework	0.91 (0.96)							0.682	0.882
9. Job/study	0.56 (0.84)							0.574	0.890
10. Expenditure	1.18 (1.02)							0.626	0.886
T1									
Full scale	9.69 (7.00)	0–26	0.653	− 0.143	8.3%	0%	0.916		

### Structural validity

The data was appropriate for factor analysis (Bartlett’s test of sphericity: *χ*^2^(45) = 607.00, *p* < 0.001; KMO statistic = 0.883) [15]. The scree plot and eigenvalues suggested a single factor, which explained less than 60% of the total variance (51.8%). However, as a two-factor solution did not result in a clear and interpretable factor structure (see Table 3), unidimensionality was assumed.

**Table 3:** Factor loadings, total variance explained, and Cronbach’s *α* of the 2-factor solution for the German version of the F-DLQI using exploratory factor analysis (EFA)

	**Factor**	
	**1**	**2**
Physical well-being	**0.905**	
Emotional impact	**0.873**	
Burden of care	**0.676**	
Expenditure	**0.642**	
People’s reaction	**0.396**	
Social life		**0.865**
Leisure activities		**0.774**
Job/study		**0.513**
Relationships		**0.424**
Housework	0.368	**0.404**
Total variance explained (in %)	51.8	9.7
Cronbach’s ***α***	0.838	0.835

Bartlett’s test of sphericity indicated that the correlation matrix was not random (*χ*^*2*^(45) = 609.13, *p* < 0.001), and the KMO statistic (0.883) was above the recommended threshold of 0.6, showing that the data was appropriate for factor analysis. The first factor had an eigenvalue of 5.179 and accounted for 51.79% of the variance; the second factor had an eigenvalue of 0.975 and accounted for 9.75% of the variance. The two factors together accounted for 61.53% of the total variance, which is above the recommended threshold of 50%, and were significantly correlated (*r* = 0.726). In line with Basra et al. [3], we decided on a unidimensional structure for the German version of the F-DLQI, given the dominance of the first factor and the ratio between the two factors being greater than the recommended ratio of 4:1 for supporting uni-dimensionality

### Reliability

Cronbach’s *α* of the F-DLQI was 0.896 (McDonald’s *ω* = 0.897), indicating good internal consistency. Item-total correlations were mostly above 0.50, and deletion of any item would not substantially increase Cronbach’s alpha (Table 2). Forty-six families (37%) filled in the second questionnaire (T1), with *M* = 17.3 days (*SD* = 10.7; range, 5–66) between the two measurements. A post hoc sample size calculation using a Shiny App [20] (expected ICC ≥ 0.75 [3], accepted lower limit of the confidence interval for ICC = 0.55, confidence interval proportion = 0.95, power = 0.8, repeated measures = 2) indicated that, using a conservative estimate, *n* = 50 would be sufficient for the present reliability testing. The ICC was 0.867 (95% CI, 0.760–0.927), indicating good test–retest reliability across the span of 2 weeks; SEm was 3.36, the MDC_individual_ score was 9.31 points, and the MDC_group_ score was 2.81.

### Construct validity

Confirming the hypothesis, significantly higher F-DLQI scores were found among the parents of moderately to severely affected children compared to those with no or mild burden (see Table 4). The F-DLQI did not discriminate between the child’s age group nor between sex.

**Table 4:** Results of ANOVAs testing for known-groups validity of F-DLQI

	***F***-DLQI score	***F***-value	***p***-value	***η***_p_^2^	Hypothesis confirmed?
GCA (< 2 vs ≥ 2)	8.48 (± 6.06) vs 11.37 (± 7.25)	*F*(1,124) = 5.77*	0.018	0.044	Yes
Age (0–2 vs 3–4 years)	9.47 (± 6.79) vs 10.96 (± 6.91)	*F*(1,124) = 1.40	0.239	0.011	No
Sex child (girls vs boys)	10.02 (± 7.38) vs 10.03 (± 6.51)	*F*(1,125) = 0.000	0.995	0.000	No

*p < 0.05

### Correlates of infants and toddlers’ HRQoL

Preliminary analysis indicated significant correlations between InToDermQoL and disease severity (GCA, BSA), the child’s current symptoms as reported by the parent, and F-DLQI. These variables were entered in the regression equation.

Only children’s current complaints (*β* = 0.083, *p* < 0.001) and F-DLQI (*β* = 0.041, *p* < 0.001) were significant correlates of InToDermQoL, explaining 76.9% of the variance (Table 5.).

**Table 5:** Correlates of infants and toddlers’ HRQoL (InToDermQoL)^†^

Variable	Unstandardized (***B***)	Standardized (*β*)	Standard error
Slope	− 0.088		0.069
BSA	0.001	0.027	0.002
GCA	− 0.020	− 0.014	0.067
Current complaints	0.083**	0.529	0.010
F-DLQI	0.041**	0.416	0.007
*R*^2^	0.769		
Corrected *R*^2^	0.760		
*F* (df = 4; 106)	88.270**		

*BSA* body surface area, *GCA* global clinical assessment (one-item measure of dermatosis severity; 0, no–mild burden; 1, moderate–very severe burden), *F-DLQI* Family Dermatology Life Quality Index, *current complaints* sum score of parent-rated items (0, not at all/never; 4, very much/always) on the child’s itch, sleeplessness, bleeding, and inflammation

†*n* = 122, as four cases were outliers and had to be removed to achieve normality

*p < 0.05; **p < 0.001

## Discussion

The results demonstrate that the German version of the F-DLQI questionnaire is a valid and reliable instrument for the assessment of HRQoL in parents of children between 0 and 4 years of age with atopic dermatitis and other skin diseases. The assumed unidimensionality of the original F-DLQI version was supported [3]. While internal consistency and test–retest reliability could be confirmed, construct validity in terms of known-group validity was only confirmed partially: the F-DLQI could discriminate between disease severities, but not between age groups and the child’s sex. In contrast to the original version [3], discriminative validity between different dermatoses could not be tested due to the uneven number of diagnoses included in this sample, with the majority of children being affected by atopic dermatitis. This and testing the responsiveness are important objectives for further validation studies that should also take into account larger and more heterogeneous samples in terms of different diagnoses.

A second contribution of this study was the examination of associations between parents’ HRQoL impairments and pediatric HRQoL. Results have shown that the child’s current complaints, such as itch and sleeplessness, and parental HRQoL, are significantly associated with the child’s HRQoL. The results confirm the importance for dermatologists and other healthcare providers, such as pediatricians, in considering the burden parents experience due to their child’s illness. This allows them to offer appropriate support options to parents as the “greater patients” at an early stage, preventing parental stress from becoming chronic and negatively affecting their children’s HRQoL. A recent meta-analysis showed that the significant negative impact of atopic dermatitis, which is the most prevalent skin disease in childhood, on caregivers’ HRQoL was mainly in the domains of family finances, caregiver burnout, and sleep impairment [30]. Another systematic review highlighted the emotional burden of family members, in particular feelings of blame, guilt, and worry, parental feelings of inadequacy in their ability to prevent the disease, negative feelings against other parents with perceived “normal” children, and fears and concerns about the impact on their child’s self-esteem and social interactions [26].

A limitation of this study is its cross-sectional design, allowing only for conclusions on bi-directional associations between sociodemographic, clinical, and parent-reported outcomes. Longitudinal studies to examine predictors of children’s HRQoL could provide more in-depth insights. Furthermore, the sample size of this study is relatively small, especially for the calculation of retest reliability. Typically, samples of *n* ≥ 50 or even ≥ 100 are recommended for reliability studies [19]. Yet, based on the sample size calculation reported above, the present sample size can still be considered adequate. Another limitation is that the majority of children in this study were diagnosed with atopic dermatitis whereas other skin diseases were underrepresented, limiting the generalizability of the findings. Hence, it is highly recommended that future studies revalidate the German version of the F-DLQI in a larger sample with more diverse dermatoses. Nonetheless, this is the first study assessing the psychometric properties of the German F-DLQI for young children 0–4 years old and the associations between a variety of variables and the child’s HRQoL, which is particularly important in terms of prevention in order to avoid chronification, and, ultimately, support a healthy childhood development. In practice, dermatologists and pediatricians can offer information and/or referral to different types of support to affected families, including self-help organizations and psychological support, but also specific structured education/prevention programs with proven effectiveness in improving health outcomes [13]. Parents’ need for comprehensive information about underlying diagnostic and therapeutic concepts might often not be fully met during medical consultations. Children and their families are challenged to cope with the difficulties caused by chronic illness. In this context, educational programs that address parents’ emotional challenges can help prevent difficulties in parent–child interaction and attachment and, in adherence to treatments, ultimately help increase the wellbeing of affected children and their families.

## Data Availability

The data that support the findings of this study are available from the corresponding author upon reasonable request.

## Abbreviations

BSA: Body surface area
EFA: Exploratory factor analysis
F-DLQI: Family dermatology life quality index
GCA: Global clinical assessment
HRQoL: Health-related quality of life
ICC: Intraclass correlation coefficient
InToDermQoL: Infants and toddlers dermatology quality of life questionnaire
MDC: Minimal detectable change
SEm: Standard errors of measurement
